# Sensitivity of cohesin–chromatin association to high-salt treatment corroborates non-topological mode of loop extrusion

**DOI:** 10.1186/s13072-021-00411-w

**Published:** 2021-07-28

**Authors:** Arkadiy K. Golov, Anastasia V. Golova, Alexey A. Gavrilov, Sergey V. Razin

**Affiliations:** 1 Institute of Gene Biology, Russian Academy of Sciences, Moscow, Russia; 2 Mental Health Research Center, Moscow, Russia; 3 Center of Life Sciences, Skolkovo Institute of Science and Technology, Moscow, Russia; 4 Center for Precision Genome Editing and Genetic Technologies for Biomedicine, Institute of Gene Biology, Russian Academy of Sciences, Moscow, Russia; 5 Faculty of Biology, M.V. Lomonosov Moscow State University, Moscow, Russia

**Keywords:** Cohesin, Loop extrusion, Topological entrapment, Chromatin folding, CTCF

## Abstract

Cohesin is a key organizer of chromatin folding in eukaryotic cells. The two main activities of this ring-shaped protein complex are the maintenance of sister chromatid cohesion and the establishment of long-range DNA–DNA interactions through the process of loop extrusion. Although the basic principles of both cohesion and loop extrusion have been described, we still do not understand several crucial mechanistic details. One of such unresolved issues is the question of whether a cohesin ring topologically embraces DNA string(s) during loop extrusion. Here, we show that cohesin complexes residing on CTCF-occupied genomic sites in mammalian cells do not interact with DNA topologically. We assessed the stability of cohesin-dependent loops and cohesin association with chromatin in high-ionic-strength conditions in G1-synchronized HeLa cells. We found that increased salt concentration completely displaces cohesin from those genomic regions that correspond to CTCF-defined loop anchors. Unsurprisingly, CTCF-anchored cohesin loops also dissipate in these conditions. Because topologically engaged cohesin is considered to be salt resistant, our data corroborate a non-topological model of loop extrusion. We also propose a model of cohesin activity throughout the interphase, which essentially equates the termination of non-topological loop extrusion with topological loading of cohesin. This theoretical framework enables a parsimonious explanation of various seemingly contradictory experimental findings.

**Supplementary Information:**

The online version contains supplementary material available at 10.1186/s13072-021-00411-w.

## Background

Cohesin is a large nuclear protein complex involved in maintaining the structure and integrity of the genome in virtually all eukaryotic cells [1, 2]. The two major functions of cohesin are chromatin spatial folding during interphase and cohesion of sister chromatids from replication until anaphase onset. Cohesin belongs to the family of SMC (structural maintenance of chromosomes) complexes, which also includes eukaryotic condensin and SMC5/6 complexes as well as various types of prokaryotic SMC complexes. The members of this ubiquitous group of ATP-driven molecular machines participate in various processes associated broadly with maintaining the control of long genomic DNA molecule 3D folding. It appears that the primordial activity of SMC complexes involved the individualization of sister chromosomes, and, thus far, each cellular division has been found to rely on one or another type of SMC complex.

**Fig. 1:**
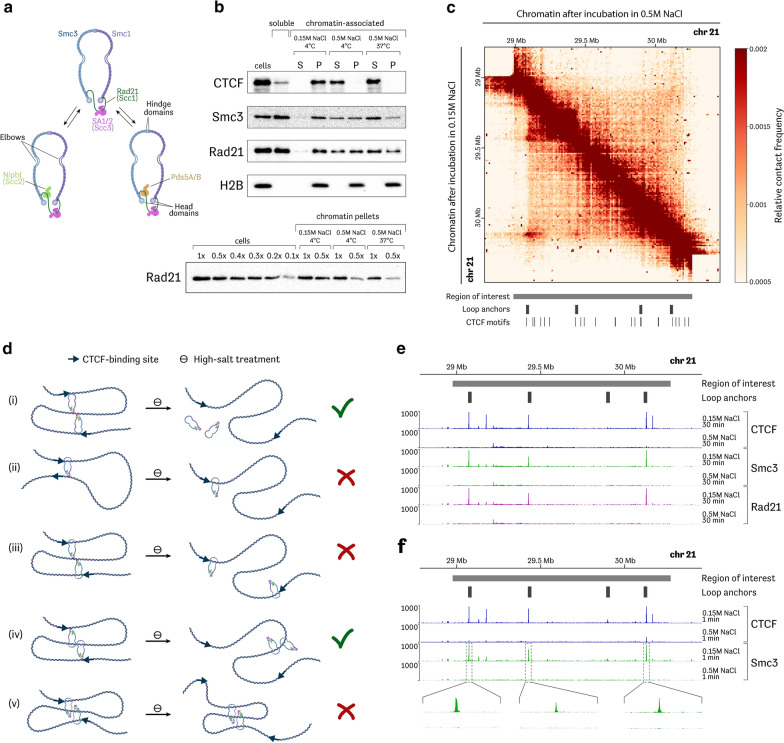
High-salt treatment causes cohesin immediate detachment from CTCF-defined binding sites and dissociation of chromatin loops. **a** Schematic of cohesin complex with stable tetrameric ring and dynamically associating HEAT-repeat regulatory subunits Scc2 and Pds5. **b** Western blots reflecting redistribution of CTCF and cohesin subunits (Smc3 and Rad21) between chromatin pellets (P) and soluble fraction (S) after treatment of permeabilized cells with either isotonic buffer or high-salt buffer. Salt-resistant histone protein H2B was used as loading control. **c** Heatmap representing chromatin contact frequencies inside the studied genomic region (hg19, chr21:28,981,189–30,260,402) in control (lower-left corner) and salt-treated (upper-right corner) nuclei. **d** Possible configurations of salt-sensitive CTCF-defined chromatin loops in terms of cohesin–DNA interaction mode. Note that only structures (i) and (iv) can be reconciled with our ChIP-seq data. **e** ChIP-seq profiles representing association of CTCF and cohesin subunits (Smc3 and Rad21) with DNA within the studied region after 30-min chromatin incubation in either control buffer or high-ionic-strength buffer. **f** ChIP-seq profiles representing association of CTCF and cohesin subunit Smc3 with DNA within the studied region after 1-min chromatin incubation in either control buffer or high-ionic-strength buffer

Despite their relative functional diversity, all SMC complexes have a highly similar and specific structural organization, which includes the triptate SMC–kleisin ring and several regulatory subunits more or less dynamically interacting with a kleisin protein [2]. Cohesin comprises an Smc1/Smc3 dimer, Rad21 (Scc1 in budding yeast) kleisin subunit and SA (Scc3) protein which is stably associated with the kleisin (Fig. 1a). SA is a large hook-shaped protein, one of the group of so-called HAWK (HEAT-repeat containing proteins associated with kleisin) proteins [3, 4]. Two other accessory HAWK proteins—Nipbl (Scc2) and Pds5—interact with kleisin dynamically. Of note, Scc2 and Pds5 compete for the same binding surface on Rad21; therefore, their association with cohesin is mutually exclusive [5–7].

It is well recognized that sister chromatid cohesion depends on the ability of cohesin to topologically entrap DNA molecules within the SMC–kleisin ring [8, 9]. There is also evidence that each cohesive cohesin ring entraps both sister chromatids [9, 10]. Such topological interaction is different from classic protein–DNA binding. The latter heavily relies on electrostatic interactions, which renders it sensitive to an increased concentration of counterions in the buffer [11]. In contrast, the topological entrapment of DNA with cohesin appears to be salt resistant [12–14]. Topological entrapment provides exceptional stability for cohesin–DNA interactions. Topologically engaged cohesin complexes could persist on DNA for hours without dissociating from it [15, 16]. The most part of cohesive complexes detach from chromatin in higher eukaryotes during prophase when cohesin rings open through disengagement of the Smc3–kleisin interface [17–19]. This process is catalyzed by Wapl (a protein that transiently interacts with Pds5), the activity of which is inhibited during G2 phase with Smc3 K112/K113 acetylation and (in vertebrates) Sororin binding [17, 20, 21].

Another important and seemingly unrelated function of cohesin is the extrusion of chromatin loops (LE) [22–24]. This activity is realized throughout interphase and, in some cases, during mitosis by a cohesin subpopulation dynamically associated with chromatin [25–27]. LE implies the ability of a cohesin complex to capture a small DNA loop by bridging two neighboring DNA sites and then to gradually increase the loop by translocating in one (one-sided LE) or both directions (two-sided LE) along DNA. In vitro data indicate that cohesin is an ATP-driven molecular machine capable of rapid LE on its own [28, 29]. Several groups have shown that cohesin extrudes loops in a two-sided manner [29, 30]. Although theoretical considerations suggest, that two-sided LE by dimers of cohesin rings should take place in living mammalian cells [31], there are contradictory reports on whether this is actually the case in in vitro models [28, 29].

In cells of Bilateralia the 11-zinc-finger transcription factor CTCF blocks the process of LE when the cohesin complex approaches DNA-bound CTCF from its N-terminal side [32, 33]. Such blocking apparently does not interfere with the translocation of cohesin (or indeed a dimer of cohesin rings) in the other direction of two-sided LE; therefore, LE proceeds in a one-sided manner until cohesin encounters another chromatin-bound CTCF. Cohesin-dependent loops with fixed CTCF-defined anchors are accumulated in the cell population as a result [23]; these phased loops can be readily detected with proximity-ligation-based methods such as Hi-C [25, 26, 34].

Several lines of circumstantial evidence suggest that cohesin interacts topologically with at least one anchor of the loop during the process of extrusion. First, the comparative stability of extruding cohesin complexes on chromatin (residence time around 5–20 min) distinguishes it from other DNA-binding proteins (typical residence time below 1 min) [15, 16]. Second, the processivity of LE is negatively regulated by the same set of proteins (Pds5 and Wapl) that participate in the disengagement of topologically bound cohesin from chromatin and cohesion disruption in early mitosis [26, 35]. Finally, it is tempting to accept a parsimonious model explaining both cohesin activities with the same basic principle of topological DNA entrapment. However recently published studies performed both in vitro and in vivo suggested that topological entrapment is dispensable for cohesin LE [9, 28, 29].

Here, to determine whether cohesin complexes mediating LE are bound to chromatin in a topological manner, we analyzed the salt-sensitivity of cohesin and CTCF-anchored DNA loops in the G1 cell cycle phase. The results support a non-topological mode of LE. Additionally, we propose a new model that describes the dynamic of loop extrusion and topological DNA entrapment in the interphase nucleus and reconciles non-topological LE with the data indicating close relationships between LE and topological DNA engagement.

## Results and discussion

To find out what proportion, if any, of cohesin complexes topologically entrap DNA molecules during the G1 phase in mammalian cells, we analyzed the possibility of extracting chromatin-bound cohesin with a high-salt solution. We synchronized HeLa cells in the G1 phase, lysed them in isotonic buffer, and incubated permeabilized cells on ice in either isotonic buffer or in a buffer containing 0.5 M NaCl. This relatively high concentration of salt should cause the extraction of most non-histone DNA-binding proteins, whereas topologically bound cohesin rings should remain associated with long chromosomal DNA molecules [12–14].

We separated extracted proteins from the insoluble material by centrifugation and assessed with western blotting the distribution of cohesin subunits Rad21 and Smc3 as well as CTCF between the fractions in different conditions. As expected, CTCF remained associated with chromatin in isotonic conditions, but was completely extracted from nuclei in a high-salt buffer (Fig. 1b, Additional file 1: Figure S1). On the other hand, approximately one half of the cohesin molecules (50–55%) were solubilized during cellular lysis in the isotonic conditions, a result that is in general agreement with previous publications [13, 36]. It is assumed that this easily solubilized fraction of cohesin roughly corresponds to a subpopulation of unbound free-diffusing cohesin molecules revealed by FRAP experiments [16, 36]. In contrast to CTCF, approximately 25–30% of cohesin complexes remain associated with chromatin even after incubation in high-ionic-strength conditions (Fig. 1b, Additional file 1: Figure S1). Recently, a salt-resistant form of cohesin DNA binding was described that does not necessarily involve true topological entrapment; this mode of cohesin–DNA interactions was referred to as a “gripping state” [37–39]. Although gripping state is salt resistant at 4 °C, it was shown that it could potentially be disrupted in high-salt buffers at higher temperatures [38]. Albeit that the gripping state seems to be short lived in vivo and could only be captured in special in vitro conditions (such as the usage of non-hydrolysable ATP analogues or ATPase-deficient cohesin complexes), we checked whether salt-resistant cohesin complexes observed in G1 cells are represented at some level by “gripping” cohesin complexes. To achieve that aim, we incubated permeabilized cells in a high-salt buffer at 37 °C and assessed the redistribution of cohesin subunits between supernatant and chromatin-associated fraction. We found that in these conditions, the proportion of solubilized cohesin increased, but a substantial fraction (10–20%) still remained associated with chromatin (Fig. 1b, Additional file 1: Figure S1). It is, therefore, likely that this portion is represented by cohesin that topologically entraps chromosomal DNA during the G1 stage of the cell cycle (i.e., before the onset of DNA replication). This notion is supported by observations made in a yeast model, where topologically engaged cohesin rings could be biochemically detected even in replication-deficient cells [9].

In the next set of experiments, we investigated whether chromatin loops generated by LE are resistant to salt extraction. With this aim, we generated 3C-seq libraries from permeabilized G1 cells incubated for 30 min in either isotonic or high-salt buffer. We chose the ~ 1 Mb region on chromosome 21 that contains several well-defined CTCF-anchored cohesin loops in HeLa cells and enriched 3C-seq libraries with ligation products from this region using the C-TALE protocol [40]. Examination of heatmaps showed that high-salt treatment caused the complete disappearance of bright spots located away from the diagonal that are believed to reflect the presence of chromatin loops (Fig. 1c, Additional file 1: Figure S2). It is, therefore, likely that in vivo generated loops are sensitive to high concentrations of salt; this behavior is similar to that of cohesin loops generated in vitro [28, 29]. The latter are disrupted along with a complete dissociation of cohesin from DNA molecules when the salt concentration increases [29]. These in vitro results were interpreted in favor of a non-topological mode of cohesin LE [29].

However, our results can be explained otherwise because the C-TALE data alone, in contrast to the results of the above-mentioned in vitro experiments, do not show whether loop-maintaining cohesin molecules remained associated with chromatin after high-salt treatment. Theoretically, loops in which cohesin molecules topologically entrap DNA can be, nonetheless, sensitive to increased ionic strength. There are several possible structures of such loops (Fig. 1d). First, the cohesin molecule can associate with CTCF loop anchors asymmetrically, with one DNA anchor entrapped in a topological manner, whereas the other is not (Fig. 1d-(ii)) (hereinafter, we will refer to loops of such structure as being semi-topological). Alternatively, each cohesin molecule of a dimer, maintaining one loop, can interact with DNA in a semi-topological manner (Fig. 1d-(iii–iv)). Two principally different configurations actually correspond to such a dimer, with either both CTCF anchors occupied by topologically bound cohesin or the other way round, with both CTCF anchors associated with the non-topologically engaged salt-sensitive pole of cohesin. Finally, our experimental settings involve comparatively prolonged incubation of nuclei in a high-salt buffer. It is possible that in such a time interval, even topologically engaged cohesin molecules can diffuse from their original CTCF anchors along DNA molecules. In this scenario, even loops that do not rely on electrostatic cohesin–DNA interactions can produce blurred and, thus, indiscernible spots in C-TALE heatmaps (Fig. 1d-(v)).

To determine which of the above-presented configurations better describes real cohesin–CTCF loops, we performed ChIP-seq to identify profiles of cohesin association with the genomic region under study (1 Mb region on chromosome 21) in control and salt-treated nuclei. We found that high-salt treatment caused the displacement of cohesin from CTCF-defined loop anchors sites, which were originally enriched in it (Fig. 1e, Additional file 1: Figure S3).

Although the extraction of CTCF by a high-salt solution should release cohesin from anchorage sites, the topologically bound cohesin is expected to reside in proximity to these sites because it has limited capacity to passively diffuse along nucleosome-bound DNA [41]. However, the possibility that topologically bound cohesin rings can passively diffuse along DNA under conditions of increased salt concentration cannot be ruled out. In particular, such diffusion can occur during the 30-min incubation in a high-salt solution performed in our experiments. To exclude this possibility we repeated the ChIP-seq experiments using a significantly shorter time of incubation in the high-salt buffer (1 min instead of 30 min). We expected to observe the preservation or, perhaps, partial flattening of cohesin peaks at the original locations after this short treatment if, indeed, cohesin remained topologically bound to DNA but started to diffuse along the chromatin fiber. However, we again registered a complete disappearance of cohesin peaks (Fig. 1f, Additional file 1: Figure S3). We also analyzed genome-wide ChIP-seq profiles of cohesin–chromatin interactions after 1-min salt treatment in G1 as well as in G2 cells (Additional file 1, Figure S4). These data confirmed that CTCF-associated cohesin rings are extremely sensitive to high-salt treatment. Interestingly, these experiments also demonstrated that regulatory genomic sequences such as promoters and enhancers in which cohesin enrichment is believed to be independent of CTCF co-occupancy also lose ChIP-seq cohesin signal after brief salt treatment. Thus, we concluded that, at least around highly positioned ChIP-detectable sites, cohesin likely does not interact with DNA topologically throughout the interphase. Overall, the ChIP-seq data support either a non-topological or semi-topological structure of CTCF-anchored chromatin loops (Fig. 1d-(i) and Fig. 1d-(iv)).

**Fig. 2:**
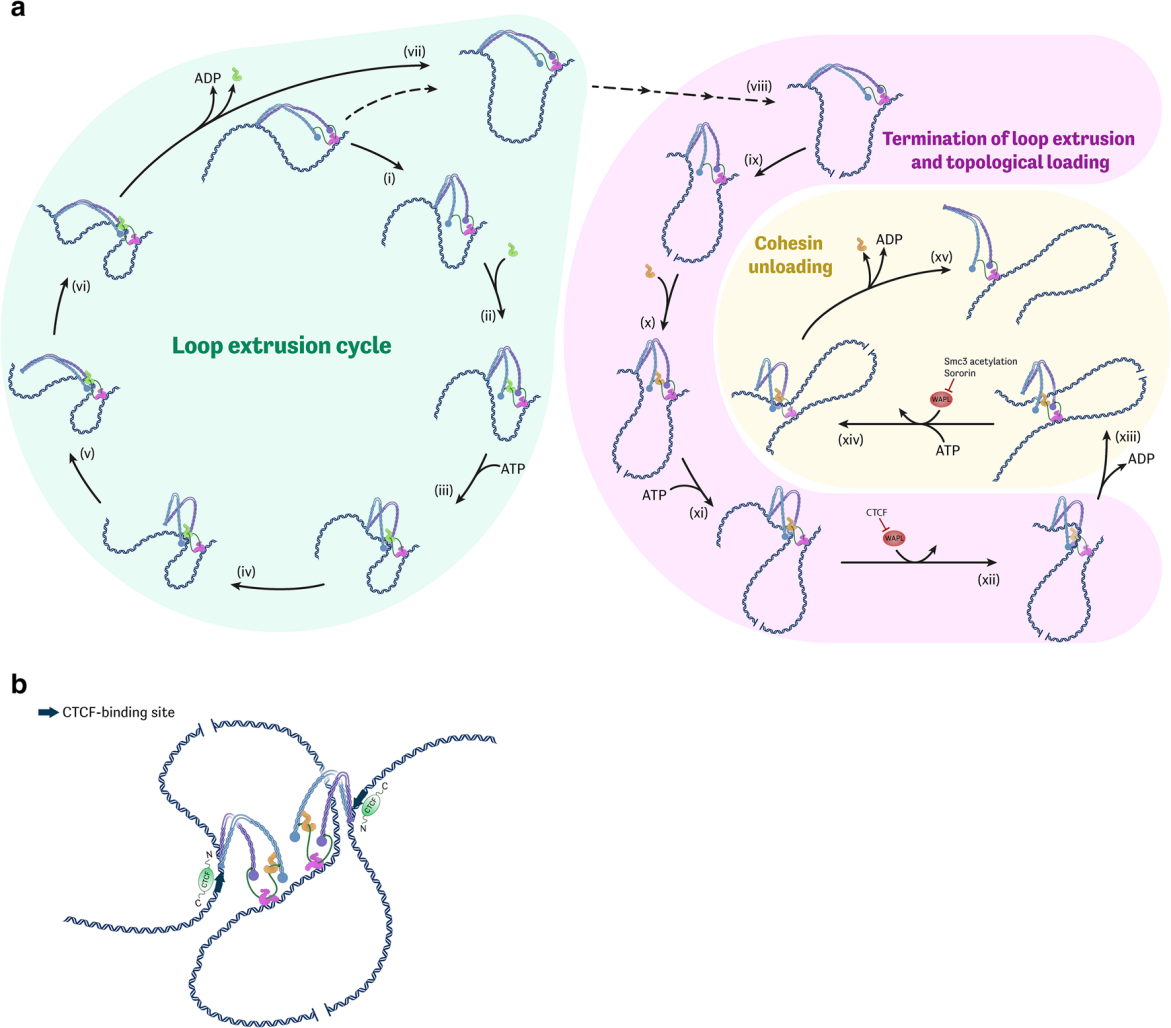
Cohesin activity during interphase. **a** Schematic of cohesin loop extrusion (LE) cycle (i–vii), Wapl-dependent termination of LE (viii–xii), and Wapl-dependent cohesin unloading (xiii–xv). Note that Scc2-catalyzed LE does not involve topological entrapment. One possible variant of cohesin structural rearrangements accompanying the LE cycle is depicted. This specific scenario involves (as was largely proposed by other authors [37, 42, 43]) cycles of cohesin bending–unbending at elbow regions of SMC coiled-coils coupled with cycles of ATP binding-hydrolysis and SMC head engagement–disengagement. During the LE cycle, the cohesin molecule constantly maintains DNA binding through one DNA-binding surface (stable anchor) while translocating along DNA with the other two DNA-interacting domains (dynamic anchor). Here, we propose that the stable DNA-binding surface is represented by Scc3, whereas DNA is transiently captured by either SMC heads and Scc2 in “gripping state” or hinge domains. LE can be terminated by Pds5–Wapl-catalyzed DNA passage through the Smc3–kleisin gate (xii), which leads to topological DNA entrapment. Note that CTCF blocks Pds5-dependent Wapl recruitment. Additional round of the gate opening (in essence a reverse reaction, catalyzed by the same protein complex) unloads cohesin from DNA and destroys the chromatin loop (xiv). The latter is inhibited in the G2 phase by Smc3 K112/113 acetylation and Sororin. **b** Hypothetical structure of CTCF-defined chromatin loop. CTCF stabilizes cohesin in the depicted conformation by promoting Pds5 binding to the complex while blocking Scc2 and Wapl recruitment. Potential propensity of asymmetrically extruding SMC complexes to form closely spaced dimmers [44] can explain formation of loops with CTCF bound to both external anchors

Our results can be accommodated by a wide range of hypothetical models of LE, in which the cohesin ring either does not physically entrap DNA at all (non-topological LE) or entraps it during only some stages of the ATP hydrolysis cycle. Below we present a model (Fig. 2a) that provides reasonable explanations for most of the apparently controversial observations. First, we suggest that LE is performed by Scc2-bound cohesin complexes in a non-topological manner. This proposal is consistent with the in vitro data on cohesin LE [28, 29] and is corroborated by our results. Additionally, topological entrapment was shown to be dispensable for cohesin translocation from the loading sites in yeast [9]. Further, we postulate that Pds5 blocks in two ways the Scc2 activity in the LE process, namely (i) Pds5 competes with Scc2 for a binding surface on Rad21 and (ii) Pds5 participates in LE termination by recruiting Wapl, which causes the temporary opening of the Smc3–kleisin gate, leading to topological DNA entrapment. This process apparently leads to the termination of loop extrusion. The suggested mechanism explains how both Pds5 and Wapl negatively regulate the processivity of LE [26, 35]. In the proposed scenario, the Pds5–Wapl complex, rather than Scc2, serves as an actual cohesin loader in vivo. Such activity has been indeed demonstrated in vitro [45]. The Scc2 in vitro loading activity shown in several studies is likely to rely on the same process of transient Smc3–kleisin gate opening. Taking into account several circumstantial pieces of evidence [14, 37, 38], it is reasonable, however, to assume that Scc2, in contrast to Pds5–Wapl, poorly catalyzes DNA passage through the Smc3–kleisin gate; such a reaction apparently requires multiple rounds of ATP hydrolysis cycle and specifically tailored conditions.

According to the proposed model (Fig. 2a), topologically loaded cohesin complexes are not able to resume loop extrusion and are, therefore, subsequently released from chromatin through an additional round of Pds5–Wapl-catalyzed Smc3–kleisin gate opening. Accordingly, the Pds5–Wapl complex mediates both the engagement of cohesin in topological interactions with DNA and disengagement from it, as previously suggested [45].

We propose that CTCF inhibits both LE progression and termination by selectively recruiting Pds5 to cohesin while preventing Wapl and Scc2 binding (Fig. 2b). Thus, CTCF sites are, in fact, locations for a temporal pausing of LE. It was, indeed, reported that CTCF N-terminal binding to cohesin inhibits LE termination by blocking Wapl binding to the ‘‘conserved essential surface’’ (CES) of SA protein [33]. Furthermore, various cohesin regulators, including Wapl, Shugoshin, Sororin and Scc2 (but not Pds5), contain the amino acid motif F/YXF involved in CTCF–CES interactions. Hence, it is reasonable to assume that CTCF binding may also interfere with Scc2 recruitment to cohesin. Pds5A was recently shown to interact with CTCF through its N-terminal domain [46]. Additionally, Pds5 knockdown data suggest a contribution of Pds5 in CTCF-dependent LE blockage [26]. Thus, it is possible that CTCF inhibits the processivity of cohesin by selectively recruiting Pds5 in place of Scc2 to the complex and also prevents loop dissociation by inhibiting Wapl activity.

Overall, the presented model implies that active cohesin LE does not involve topological DNA entrapment and that LE termination is catalyzed by the Pds5–Wapl complex and is associated with cohesin topological loading and subsequent release. Such a hypothetical framework reconciles non-topological LE with the fact that both Pds5 and Wapl, primarily recognized as unloading factors, negatively regulate the processivity of LE. Released cohesin rings can be involved in new rounds of LE. However, a time gap exists between LE termination and the release of cohesin from chromatin. This gap explains the existence of topologically loaded cohesin rings during the G1 phase reported in our study and in previous publications [9, 13]. This subpopulation of engaged rings can be stabilized on chromosomes during the S phase by Smc3 K112/113 acetylation and Sororin recruitment, which block Wapl-dependent cohesin release (Fig. 2a) [47, 48].

## Conclusions

Here, we showed that a small but substantial subpopulation of cohesin complexes is associated with chromatin in a salt-resistant manner during the G1 phase of the cell cycle in mammalian cells. However, cohesin association with CTCF-bound genomic regions as well as CTCF-defined loops is sensitive to high-salt treatment. We suppose that these results, in conjunction with previously published data on cohesin structure and activity, are in better agreement with a non-topological mode of LE. We also proposed a parsimonious model of cohesin activity during interphase that takes into account many experimental observations and reconciles non-topological LE with the crucial role of cohesin-releasing factors in the regulation of LE processivity.

## Methods

### Cell culture and synchronization

Human HeLa cells were cultured in a DMEM medium supplemented with 10% FBS, 100 U/ml penicillin and 100 U/ml streptomycin at 37 °C in 5% CO_2_ in a humidified atmosphere. For G1 synchronization, cells were treated with 2 mM thymidine for 20 h, washed twice with DPBS, and released into a complete medium. After 6 h, nocodazole (Sigma) was added (100 ng/ml) for 8 h. Mitotic cells were collected by shake-off and centrifugation. Cells were washed twice with DPBS and released into a fresh complete medium. For C-TALE and ChIP-seq experiments, mitotic cells were seeded in poly-L-lysine-coated dishes. For coating, dish bottoms were covered with 0.1 mg/mL solution of poly-L-lysine (Sigma, P6282) in DPBS and incubated for 1 h at room temperature; after the solution was discarded, dishes were rinsed twice with DPBS and dried for 45 min under the hood. G1 cells were harvested for experiments after 5 h of release from mitotic arrest. For late S/G2 synchronization, cells were seeded in poly-L-lysine-coated dishes, treated with 2 mM thymidine for 17 h, released for 10 h from the block, then treated with thymidine for additional 14 h and released once again into a fresh medium. Each time before release into thymidine-free medium cells were washed twice with DPBS. Late S/G2 cells were harvested for experiments after 6 h of release from the second thymidine block.

### Lysate preparation and immunoblotting

Approximately 5 mln G1 cells were harvested with 0.05% trypsin–EDTA solution and centrifuged; the pellet was resuspended in 1 mL of PBS. One-quarter of the suspension was centrifuged and the cellular pellet was lysed in 600 uL of ice-cold RIPA buffer (50 mM Tris–HCl pH 8.0, 150 mM NaCl, 1% Triton X-100, 0.5% sodium deoxycholate, 0.1% SDS) supplemented with protease inhibitors (Bimake, B14001). The cellular lysate was stored in ice until shearing (see below). The remaining part of the cellular suspension was centrifuged and the pellet was resuspended in 750 uL of ice-cold isotonic lysis buffer (10 mM Hepes pH 8.0, 145 mM NaCl, 1.5 mM MgCl_2_, 1% NP-40, 1 × protease inhibitors). The permeabilization was performed on ice for 10 min, and the suspension was then centrifuged. The supernatant was collected and diluted with an equal volume of isotonic lysis buffer (supernatant 1). The pellet was thoroughly resuspended in 50 ul of ice-cold isotonic buffer (10 mM Hepes pH 8.0, 145 mM NaCl, 1.5 mM MgCl_2_, 0.5% NP-40, 1 × protease inhibitors), and the suspension was separated in three equal parts. One aliquot was diluted with 470 uL of the same isotonic buffer, the other two—with 470 uL of ice-cold high-salt buffer (10 mM Hepes pH 8.0, 500 mM NaCl, 1.5 mM MgCl_2_, 0.5% NP-40, 1 × protease inhibitors). The isotonic aliquot and one of high-salt aliquots were incubated at 4 °C. The other high-salt aliquot was incubated in a preheated thermoblock at 37 °C. All suspensions were occasionally agitated. After a 30-min incubation chromatin pellets were separated from solubilized material with centrifugation at 20,000*g* for 5 min. Each of the three chromatin pellets were lysed in 600 uL of ice-cold RIPA buffer supplemented with protease inhibitors. Supernatants were cleared up with an additional round of centrifugation at 20,000*g* for 5 min (supernatants 2–4). Solubilized proteins from all four generated supernatants (supernatants 1–4) were subjected to three rounds of concentration in 30-kDa Amicon filter columns (Millipore, UFC503096) with a subsequent reconstituting volume with RIPA buffer; after the final round of concentration, material from each supernatant was brought to 600 uL with RIPA buffer supplemented with protease inhibitors. Cellular and chromatin lysates were sheared with a VirSonic 100 cell disrupter. DNA was isolated from 20-uL aliquots of sonicated material from each pellet (cellular or chromatin). DNA quantities were measured with a Qubit fluorometer and concentrations in original lysates were calculated. Each sample (both lysates from pellets and supernatant samples) was diluted with RIPA to obtain final solutions such that each uL would contain or correspond to (in the case of supernatant samples) approximately 10 ng of DNA. Final samples were stored at 4 °C for several days until they were loaded into sodium dodecyl sulfate-polyacrylamide gel.

Polyacrylamide gel electrophoresis and immunoblotting were performed as described in [49].

### C-TALE and ChIP-seq

Chromatin for C-TALE and ChIP-seq experiments was prepared as follows. G1-synchronized HeLa cells in 10-cm dishes were washed once with PBS and dishes were cooled on ice for several minutes. 5 mL of ice-cold isotonic lysis buffer (10 mM Hepes pH 8.0, 145 mM NaCl, 1.5 mM MgCl_2_, 1% NP-40, 1 × protease inhibitors) were added to each dish, cells were permeabilized for 10 min on ice, then the buffer was removed. 5 mL of either ice-cold isotonic buffer (10 mM Hepes pH 8.0, 145 mM NaCl, 1.5 mM MgCl_2_, 0.5% NP-40, 1 × protease inhibitors) or high-salt buffer (10 mM Hepes pH 8.0, 500 mM NaCl, 1.5 mM MgCl_2_, 0.5% NP-40, 1 × protease inhibitors) was added to permeabilized cells. Dishes were incubated at 4 °C for 30 min. In one series of ChIP-seq experiments, the incubation time was shortened to 1 min. After incubation, permeabilized cells were rinsed three times with 5 mL of ice-cold wash buffer (10 mM Hepes pH 8.0, 145 mM NaCl, 1.5 mM MgCl_2_). Each washing was performed on ice and lasted for 10 min. After the final portion of wash buffer was discarded, chromatin was fixed for 10 min at room temperature with 9 ml of 2% formaldehyde solution in the wash buffer. Fixation was quenched by adding 1 ml of 2 M glycine for 10 min. Fixed chromatin was washed once with PBS and scraped in 7 mL of ice-cold Farnham lysis buffer (5 mM Hepes pH 8.0, 85 mM KCl, 0.5% NP-40) supplemented with protease inhibitors. Chromatin was collected by centrifugation at 1,000*g* for 5 min at 4 °C.

For C-TALE experiments, chromatin pellets were resuspended in the restriction digestion buffer, and then C-TALE was performed essentially as described previously [40]. Restriction endonuclease NlaIII was used for DNA digestion. An equimolar mix of BAC DNA isolated from 7 clones—RP11-690G6, RP11-619G21, RP11-30C13, RP11-297L18, RP11-916H5, RP11-1054D23, RP11-791E20—was used for probe preparation. The probes covered the 1.3-Mb region of the human genome on chromosome 21: 28,981,189–30,260,402 (hg19) representing the region of interest. Experiments for both studied conditions (control chromatin and high-salt-treated chromatin) were performed in three biological replicates. C-TALE libraries were sequenced (PE150) with Illumina NextSeq and HiSeq 2500 platforms. Reads were mapped to the 4-Mb fragment of the genome (hg19, chr21:27,922,688–32,028,897), in which the region of interest was embedded, using Bowtie2 v2.3.5 [50]. The data were processed using the hiclib pipeline [51]. Statistics for the C-TALE data processing can be found in Additional file 2: Table S1. 10 kb-binned HDF5 files were converted to cool format matrices using cooler v0.8.7. Data from three biological replicates for each experimental condition (control and high-salt-treated chromatin) were merged in two matrices which were then iteratively normalized using a publicly available script [52] (“–mult_factor 2” option). Weights in a few poorly covered bins were reduced to NA during the normalization procedure; the rhdf5 (2.34.0) R package was used to manually replace these NA weights with the values of the highest weight found in each matrix. C-TALE heatmaps were visualized in matplotlib v3.2.1 with the “cooler show” command. Pairwise stratum-adjusted correlation coefficients (SCC) between matrices were calculated with hicrep (1.12.2) R package. Hierarchical clustering and visualizations of SCC heatmap and dendrogram were performed with pheatmap (1.0.12) R package.

For ChIP-seq experiments, fixed chromatin pellets were resuspended in 600 uL of ice-cold RIPA buffer supplemented with protease inhibitors. Chromatin was sheared on ice with a VirSonic 100 cell disrupter with 10 30-s pulses on “15” power setting, separated with 3-min periods of recovery. Input DNA was isolated from 1/10 aliquots of sonicated samples. Sheared chromatin from approximately 1 mln cells and 1 ug of antibodies (against either CTCF, Smc3, or Rad21) were used in each immunoprecipitation reaction. Chromatin immunoprecipitation was performed as described in the Abcam X-ChIP manual [53] with minor modifications. 25 ul of protein A/G magnetic beads (Thermo Scientific, 26,162) was used per reaction instead of agarose beads; thus, a magnet was used instead of a centrifuge to reclaim beads. Beads were blocked with 1% BSA solution in RIPA overnight, meanwhile chromatin was incubated with antibodies. BSA-blocked beads were mixed with antibody-bound chromatin for 6 h. Immunoprecipitated DNA was separated from beads by overnight treatment with proteinase K (Thermo Scientific, EO0491) in PBS supplemented with 1% SDS at 65 °C. The solution was then cleared from beads with a magnet, and DNA was isolated with standard phenol–chloroform extraction and ethanol precipitation. Sequencing libraries from both immunoprecipitated DNA and inputs were generated as described previously [40]. In a subset of “enriched ChIP-seq” experiments the same probe set as in C-TALE was used for the enrichment of ChIP-seq libraries with fragments from the chosen genomic region [40]. ChIP-seq libraries were sequenced (PE100) with Illumina NovaSeq 6000 and HiSeq 4000 platforms. Each immunoprecipitation experiment was performed in two biological replicates.

Reads from the enriched ChIP-seq libraries were mapped to the 4 Mb fragment of the genome (hg19, chr21:27,922,688–32,028,897), in which the region of interest (hg19, chr21: 28,981,189–30,260,402) was embedded, using Bowtie2 v2.3.5 [50]. Illumina TruSeq adapters were trimmed with cutadapt v1.15 [54]. Read pairs with at least one non-uniquely mapped read as well as PCR and optical duplicates were filtered using samtools v1.7. Bedtools v2.25.0 [55] and bedGraphToBigWig utility (from UCSC) were used to generate bigwig coverage files. Statistics for the ChIP-seq data processing can be found in Additional file 2: Table S2. Bigwig files were scaled to 1 mln uniquely mapped read pairs with deeptools v3.4.3 [56]. Corresponding input bigwig files were subtracted from each immunoprecipitation bigwig file and then mean profiles were calculated from pairs of biological replicates. Both operations were performed with deeptools v3.4.3. ChIP-seq bar charts were visualized with the IGV v2.8.0 desktop browser [57]. Pairwise Pearson correlations and hierarchical clustering were calculated and visualized with deeptools v3.4.3.

Reads from genome-wide ChIP-seq libraries (we used only the first read from each pair) were mapped to the hg19 reference human genome using Bowtie2 v2.3.5. Illumina TruSeq adapters were trimmed with cutadapt v1.15. Non-uniquely mapped reads as well as PCR and optical duplicates were filtered using samtools v1.7. Statistics for the genome-wide ChIP-seq data processing can be found in Additional file 2: Table S3. RPKM-normalized bigwig files were generated; the average bigwig profile for each pair of biological replicates was created from normalized bigwig files. Both operations were performed with deeptools v3.4.3. ChIP-seq heatmaps and density profiles were built, pairwise Pearson correlations and hierarchical clustering were calculated and visualized with deeptools v3.4.3.

Coordinates of CTCF-bound sites used for plotting of ChIP-seq heatmap were derived as follows. CTCF peaks were called in both replicates of control CTCF ChIP-seq with MACS2 v2.1.1 [58]. We used input data from our previous publication [59] as control profiles for peak calling. Peaks falling into ENCODE-blacklisted regions were discarded and finally peaks reproducibly detected in both replicates were annotated as CTCF-bound sites. Promoters used for plotting of ChIP-seq heatmap were downloaded from UCSC Table Browser (hg19, UCSC genes, knownGene, bed, “Upstream by 10 bases”). Genomic coordinates of enhancers active in HeLa cells were taken from [60]. Only 2,000 promoters and 2,000 enhancers with the highest SMC3 ChIP-seq signal in G2 control dataset were used for heatmap plotting.

### Motif search

CTCF motifs were annotated inside the region of interest (hg19, chr21:28,981,189–30,260,402) with HOMER package v4.11 [61] (findMotifs.pl command with “-find ctcf.motif” option).

### Antibodies

Antibodies against histone H2B (Active Motif, 61,037), CTCF (Active Motif, 61,311), cohesin subunits Rad21 (Abcam, ab992) and Smc3 (Abcam, ab9263), as well as secondary horseradish peroxidase-conjugated antibodies against mouse IgG (GE Healthcare, NA931VS) and against rabbit IgG (GE Healthcare, NA934VS) were used.

## Supplementary Information

**Additional file 1:** **Figure S1.** Western blots reflecting redistribution of CTCF and cohesin subunit Smc3 between chromatin pellets and soluble fraction after treatment of permeabilized cells with either isotonic buffer or high-salt buffer. Samples for the depicted blots represent a biological replicate of the experiment shown in Fig. 1b. Only material from pellets is analyzed. Note the almost complete extraction of CTCF with high-salt buffers and salt-resistant subpopulation of cohesin (Smc3). Histone protein H2B was used as loading control. **Figure S2.** Salt-induced dissociation of chromatin loops assessed with C-TALE is highly reproducible. **a** Heatmaps representing chromatin contact frequencies inside the studied genomic region (hg19, chr21:28,981,189–30,260,402) in three biological replicates of control (upper row) and salt-treated (lower row) nuclei. **b** Heatmap showing pairwise similarity between all C-TALE experiments calculated using stratum-adjusted correlation coefficient (SCC). Dendrogram depicts the result of hierarchical clustering analysis based on the similarity scores. **Figure S3.** Salt-induced dissociation of CTCF and cohesin from chromatin is highly reproducible. ChIP-seq profiles representing association of CTCF (**a**), Smc3 (**b**) and Rad21 (**c**) with DNA within the studied region (hg19, chr21:28,981,189–30,260,402) in each individual replicate and heatmaps showing pairwise Pearson correlations between all the experiments. **Figure S4.** Genome-wide salt-sensitivity of cohesin in highly positioned sites throughout the interphase. **a** ChIP–seq heatmaps and density profiles of the cohesin subunit Smc3 and CTCF in CTCF-bound sites, SMC3-occupied promoters and enhancers. Data for control and 1-min salt-treated chromatin in asynchronous (CTCF), G1- and G2-synchronized (Smc3) HeLa cells are presented. **b** Heatmaps showing pairwise Pearson correlations between genome-wide ChIP-seq experiments and dendrograms resulted from a hierarchical clustering analysis.

**Additional file 2: Table S1.** C-TALE sequencing and processing statistics. **Table S2.** Enriched ChIP-seq sequencing and processing statistics. **Table S3.** Genome-wide ChIP-seq sequencing and processing statistics.

## Data Availability

The datasets supporting the conclusions of this article are available from the Gene Expression Omnibus database under the accession number GSE166387 [62].
