# Supplementary material for: Sensitivity of cohesin–chromatin association to high-salt treatment corroborates non-topological mode of loop extrusion
Source: Epigenetics Chromatin. 2021 Jul 28;14:36. doi: 10.1186/s13072-021-00411-w (PMC8320178; doi:10.1186/s13072-021-00411-w)
Supplement: Supplementary file 1 — Additional file 1: Figure S1. Western blots reflecting redistribution of CTCF and cohesin subunit Smc3 between chromatin pellets and soluble fraction after treatment of permeabilized cells with either isotonic buffer or high-salt buffer. Samples for the depicted blots represent a biological replicate of the experiment shown in Fig. 1b. Only material from pellets is analyzed. Note the almost complete extraction of CTCF with high-salt buffers and salt-resistant subpopulation of cohesin (Smc3). Histone protein H2B was used as loading control. Figure S2. Salt-induced dissociation of chromatin loops assessed with C-TALE is highly reproducible. a Heatmaps representing chromatin contact frequencies inside the studied genomic region (hg19, chr21:28,981,189–30,260,402) in three biological replicates of control (upper row) and salt-treated (lower row) nuclei. b Heatmap showing pairwise similarity between all C-TALE experiments calculated using stratum-adjusted correlation coefficient (SCC). Dendrogram depicts the result of hierarchical clustering analysis based on the similarity scores. Figure S3. Salt-induced dissociation of CTCF and cohesin from chromatin is highly reproducible. ChIP-seq profiles representing association of CTCF (a), Smc3 (b) and Rad21 (c) with DNA within the studied region (hg19, chr21:28,981,189–30,260,402) in each individual replicate and heatmaps showing pairwise Pearson correlations between all the experiments. Figure S4. Genome-wide salt-sensitivity of cohesin in highly positioned sites throughout the interphase. a ChIP–seq heatmaps and density profiles of the cohesin subunit Smc3 and CTCF in CTCF-bound sites, SMC3-occupied promoters and enhancers. Data for control and 1-min salt-treated chromatin in asynchronous (CTCF), G1- and G2-synchronized (Smc3) HeLa cells are presented. b Heatmaps showing pairwise Pearson correlations between genome-wide ChIP-seq experiments and dendrograms resulted from a hierarchical clustering analysis. [file 13072_2021_411_MOESM1_ESM.pdf]

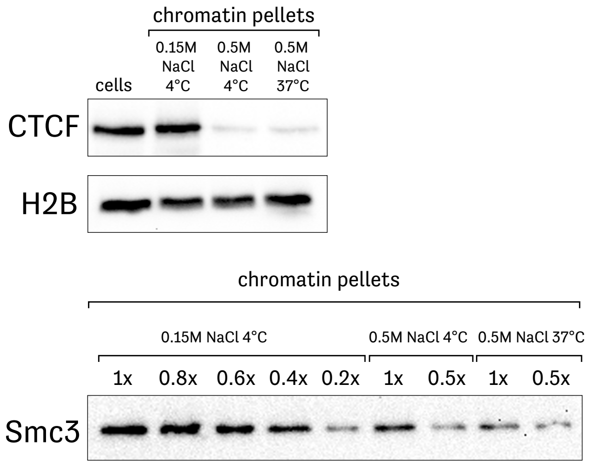

Figure S1.

Western blots reflecting redistribution of CTCF and cohesin subunit Smc3 between chromatin pellets and soluble fraction after treatment of permeabilized cells with either isotonic buffer or high-salt buffer.

Samples for the depicted blots represent a biological replicate of the experiment shown in Fig.1b. Only material from pellets is analyzed. Note the almost complete extraction of CTCF with high-salt buffers and salt-resistant subpopulation of cohesin (Smc3). Histone protein H2B was used as loading control.

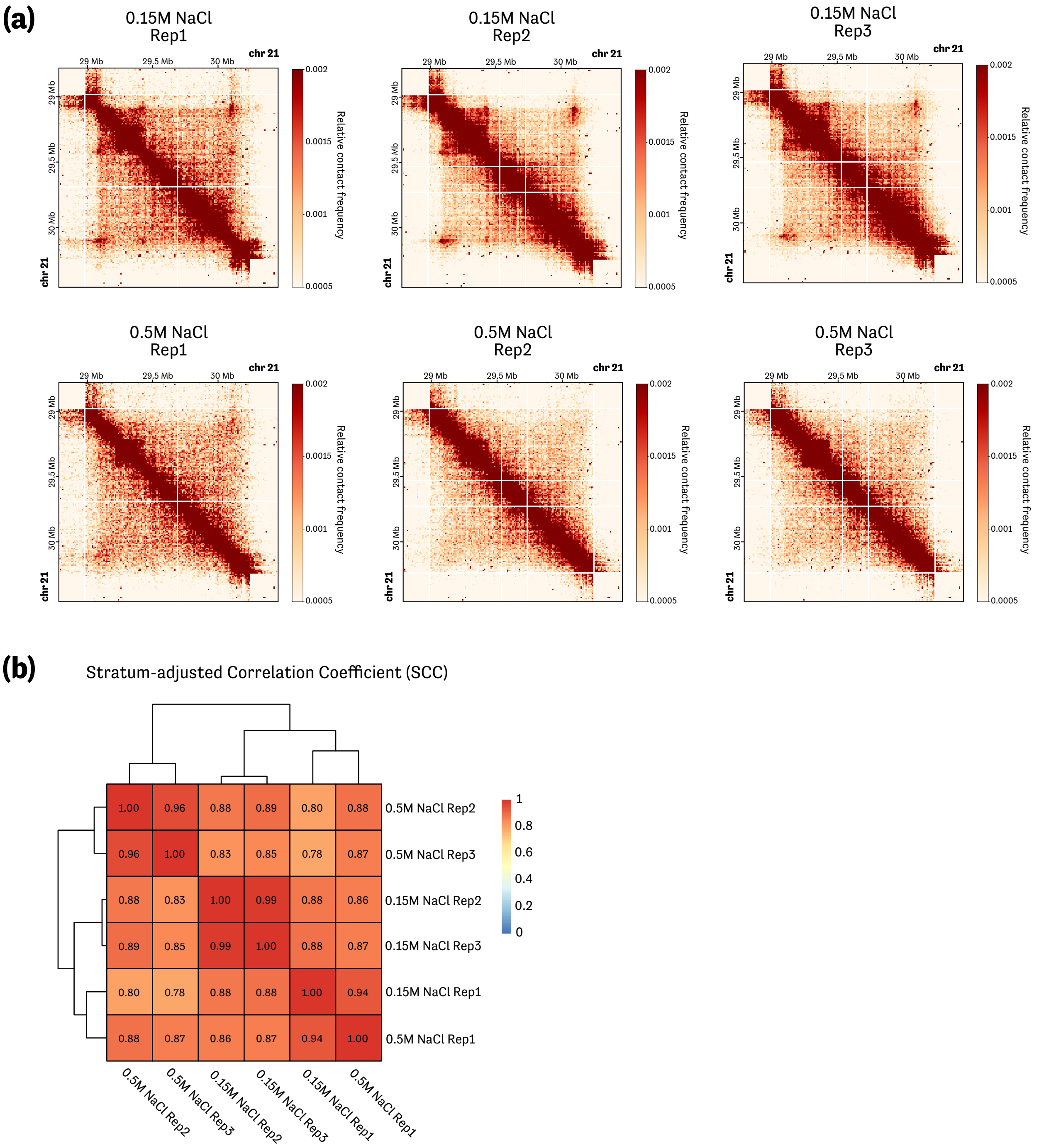

Figure S2.

Salt-induced dissociation of chromatin loops assessed with C-TALE is highly reproducible.
a Heatmaps representing chromatin contact frequencies inside the studied genomic region (hg19, chr21:28,981,189-30,260,402) in three biological replicates of control (upper row) and salt-treated (lower row) nuclei. **b** Heatmap showing pairwise similarity between all C-TALE experiments calculated using stratum-adjusted correlation coefficient (SCC). Dendrogram depicts the result of hierarchical clustering analysis based on the similarity scores.

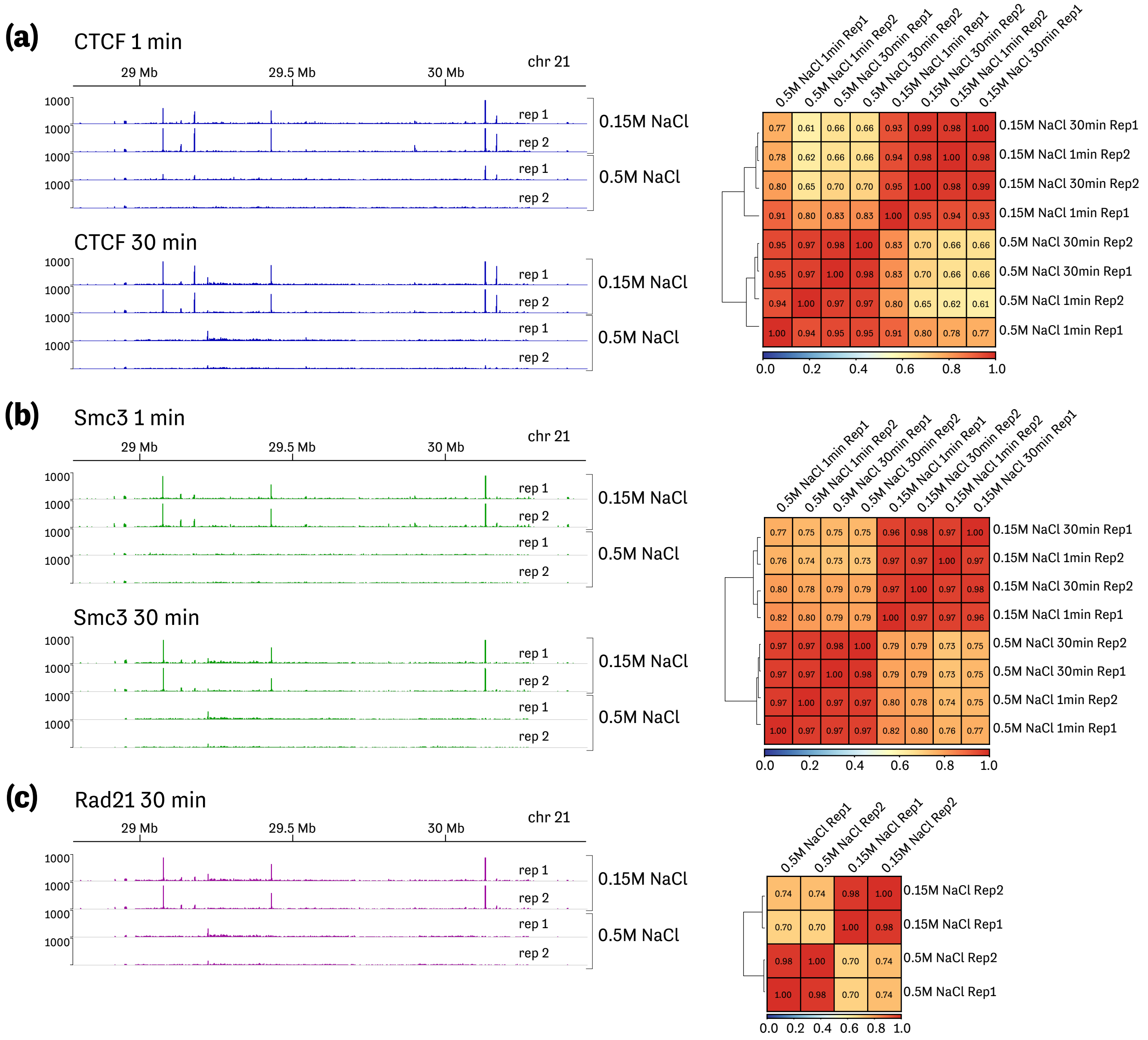

Figure S3. Salt-induced dissociation of CTCF and cohesin from chromatin is highly reproducible.

ChIP-seq profiles representing association of CTCF **(a)**, Smc3 **(b)** and Rad21 **(c)** with DNA within the studied region (hg19, chr21:28,981,189-30,260,402) in each individual replicate and heatmaps showing pairwise Pearson correlations between all the experiments.

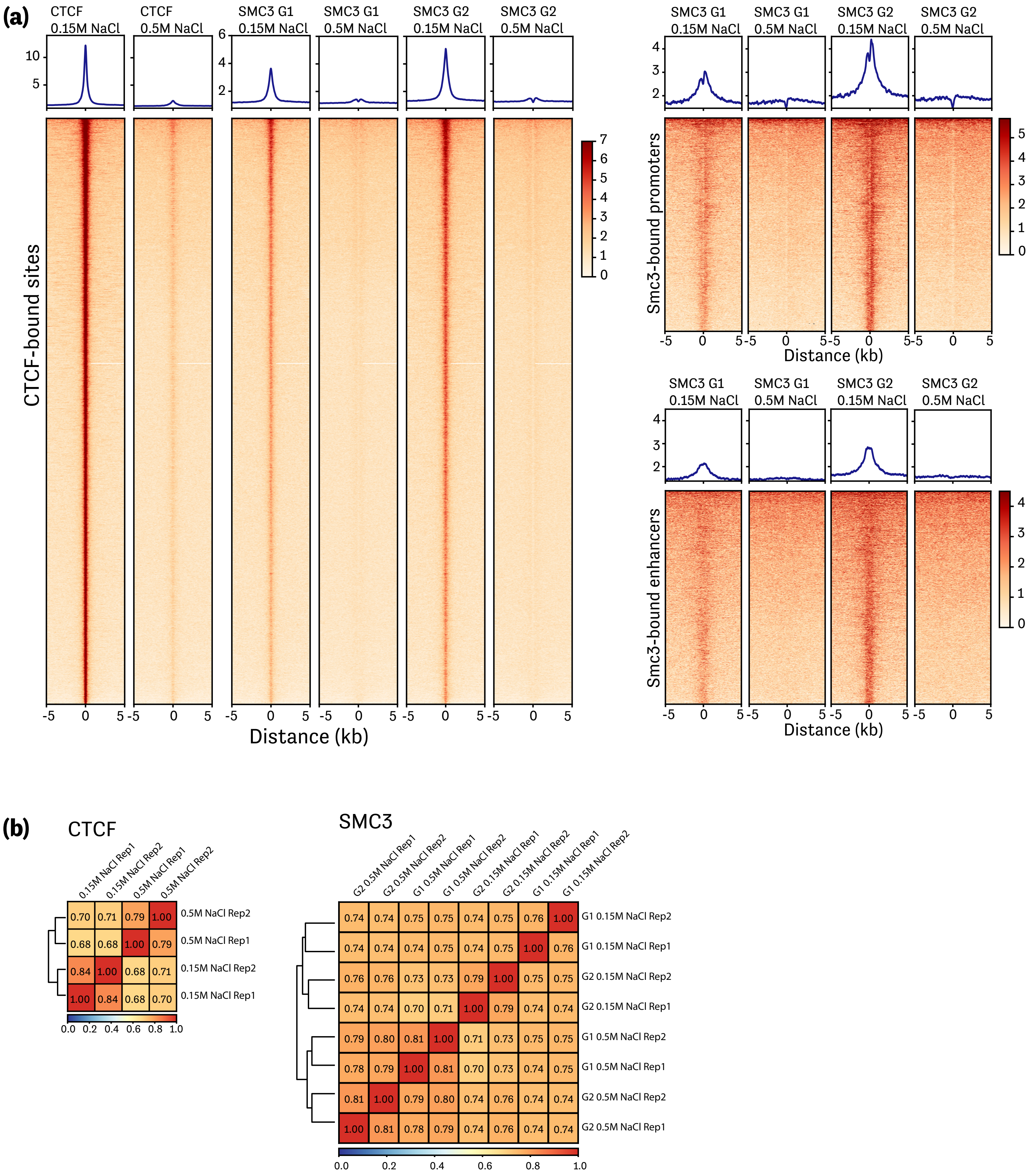

Figure S4. Genome-wide salt-sensitivity of cohesin in highly-positioned sites throughout the interphase.

a ChIP–seq heatmaps and density profiles of the cohesin subunit Smc3 and CTCF in CTCF-bound sites, SMC3-occupied promoters and enhancers. Data for control and 1-min salt-treated chromatin in asynchronous (CTCF), G1- and G2-synchronized (Smc3) HeLa cells are presented. **b** Heatmaps showing pairwise Pearson correlations between genome-wide ChIP-seq experiments and dendrograms resulted from a hierarchical clustering analysis.
