# Supplementary material for: Sensitivity of cohesin–chromatin association to high-salt treatment corroborates non-topological mode of loop extrusion
Source: Epigenetics Chromatin. 2021 Jul 28;14:36. doi: 10.1186/s13072-021-00411-w (PMC8320178; doi:10.1186/s13072-021-00411-w)
Supplement: Supplementary file 2 — Additional file 2: Table S1. C-TALE sequencing and processing statistics. Table S2. Enriched ChIP-seq sequencing and processing statistics. Table S3. Genome-wide ChIP-seq sequencing and processing statistics. [file 13072_2021_411_MOESM2_ESM.docx]

**Supplementary tables:**

**Table S1.** C-TALE sequencing and processing statistics

| **Sample** | **Number of read pairs** | | | |
| --- | --- | --- | --- | --- |
|  | **raw** | **mapped**  **(both mate reads)** | **valid pairs before filtering** | **unique valid pairs after filtering** |
| **CTALE_control_1** | 11,777,302 | 4,391,119 | 620,620 | 296,024 |
| **CTALE_control_2** | 18,660,528 | 8,617,441 | 1,519,622 | 674,087 |
| **CTALE_control_3** | 17,420,499 | 7,884,132 | 1,277,029 | 536,948 |
| **CTALE_salt_1** | 20,611,939 | 3,834,042 | 480,633 | 231,430 |
| **CTALE_salt_2** | 13,681,864 | 5,765,398 | 803,334 | 352,634 |
| **CTALE_salt_3** | 18,123,950 | 7,840,440 | 895,634 | 332,061 |

**Table S2.** Enriched ChIP-seq sequencing and processing statistics

| **Sample** | **Number of read pairs** | | |
| --- | --- | --- | --- |
|  | **raw** | **mapped**  **(both mate reads)** | **unique** |
| **ChIP_1min_input_control_1** | 4,028,391 | 995,769 | 775,231 |
| **ChIP_1min_input_control_2** | 3,905,615 | 938,409 | 731,019 |
| **ChIP_1min_input_salt_1** | 5,274,484 | 1,269,879 | 946,612 |
| **ChIP_1min_input_salt_2** | 4,813,205 | 1,217,751 | 918,224 |
| **ChIP_1min_CTCF_control_1** | 4,044,108 | 803,081 | 606,644 |
| **ChIP_1min_CTCF_control_2** | 4,562,380 | 1,008,915 | 676,053 |
| **ChIP_1min_CTCF_salt_1** | 4,283,385 | 1,049,207 | 733,957 |
| **ChIP_1min_CTCF_salt_2** | 4,954,562 | 1,276,476 | 973,222 |
| **ChIP_1min_Smc3_control_1** | 3,965,801 | 772,953 | 557,853 |
| **ChIP_1min_Smc3_control_2** | 3,402,620 | 667,066 | 479,500 |
| **ChIP_1min_Smc3_salt_1** | 4,836,275 | 1,071,168 | 804,210 |
| **ChIP_1min_Smc3_salt_2** | 5,709,959 | 1,318,239 | 979,616 |
| **ChIP_30min_input_control_1** | 3,461,126 | 1,262,669 | 994,440 |
| **ChIP_30min_input_control_2** | 4,949,695 | 1,567,422 | 1,093,228 |
| **ChIP_30min_input_salt_1** | 4,280,107 | 1,622,571 | 1,244,469 |
| **ChIP_30min_input_salt_2** | 5,101,719 | 1,722,070 | 1,245,755 |
| **ChIP_30min_CTCF_control_1** | 5,015,998 | 1,217,423 | 774,118 |
| **ChIP_30min_CTCF_control_2** | 4,517,698 | 1,289,284 | 813,884 |
| **ChIP_30min_CTCF_salt_1** | 6,642,253 | 1,816,290 | 1,046,950 |
| **ChIP_30min_CTCF_salt_2** | 6,867,664 | 2,077,149 | 1,054,693 |
| **ChIP_30min_Smc3_control_1** | 4,130,380 | 1,140,399 | 704,872 |
| **ChIP_30min_Smc3_control_2** | 5,149,328 | 1,255,375 | 799,590 |
| **ChIP_30min_Smc3_salt_1** | 6,997,939 | 2,091,598 | 1,384,204 |
| **ChIP_30min_Smc3_salt_2** | 8,030,148 | 2,421,233 | 1,542,284 |
| **ChIP_30min_Rad21_control_1** | 3,923,253 | 1,125,194 | 729,827 |
| **ChIP_30min_Rad21_control_2** | 5,482,466 | 1,433,873 | 864,354 |
| **ChIP_30min_Rad21_salt_1** | 6,327,481 | 1,744,341 | 1,175,482 |
| **ChIP_30min_Rad21_salt_2** | 7,069,804 | 2,148,215 | 1,390,909 |

**Table S3.** Genome-wide ChIP-seq sequencing and processing statistics

| **Sample** | **Number of reads** | |
| --- | --- | --- |
|  | **raw** | **mapped, unique** |
| **ChIP_gw_CTCF_control_1** | 35,593,356 | 23,116,253 |
| **ChIP_gw_CTCF_control_2** | 36,539,701 | 25,365,074 |
| **ChIP_gw_CTCF_salt_1** | 46,099,981 | 27,863,652 |
| **ChIP_gw_CTCF_salt_2** | 39,250,070 | 24,335,816 |
| **ChIP_gw_Smc3_G1_control_1** | 31,730,413 | 20,927,795 |
| **ChIP_gw_Smc3_G1_control_2** | 32,870,959 | 21,663,055 |
| **ChIP_gw_Smc3_G1_salt_1** | 38,016,583 | 25,451,338 |
| **ChIP_gw_Smc3_G1_salt_2** | 45,760,237 | 30,814,510 |
| **ChIP_gw_Smc3_G2_control_1** | 29,555,072 | 18,827,045 |
| **ChIP_gw_Smc3_G2_control_2** | 32,750,269 | 21,300,464 |
| **ChIP_gw_Smc3_G2_salt_1** | 40,068,252 | 26,703,739 |
| **ChIP_gw_Smc3_G2_salt_2** | 44,837,609 | 30,482,798 |
